# β-Elemene Suppresses Obesity-Induced Imbalance in the Microbiota-Gut-Brain Axis

**DOI:** 10.3390/biomedicines9070704

**Published:** 2021-06-22

**Authors:** Yingyu Zhou, Wanyi Qiu, Yimei Wang, Rong Wang, Tomohiro Takano, Xuyang Li, Zhangliang Zhu, Haruyo Nakajima-Adachi, Masaru Tanokura, Satoshi Hachimura, Takuya Miyakawa

**Affiliations:** 1 Department of Applied Biological Chemistry, Graduate School of Agricultural and Life Sciences, The University of Tokyo, Bunkyo-ku, Tokyo 113-8657, Japan; zhouyingyu_work@outlook.com (Y.Z.); wanyichiu17@gmail.com (W.Q.); wym19958287@yahoo.co.jp (Y.W.); wangronglily@gmail.com (R.W.); ttakano@niid.go.jp (T.T.); lixuyang1215@yahoo.co.jp (X.L.); zlzhu@vip.163.com (Z.Z.); 2 Research Center for Food Safety, Graduate School of Agricultural and Life Sciences, The University of Tokyo, Bunkyo-ku, Tokyo 113-8657, Japan; aryu@mail.ecc.u-tokyo.ac.jp; 3 College of Biotechnology, Tianjin University of Science and Technology, Tianjin 300457, China

**Keywords:** obesity, inflammation, central nervous system, gut bacteria, pearson correlations

## Abstract

As a kind of metabolically triggered inflammation, obesity influences the interplay between the central nervous system and the enteral environment. The present study showed that β-elemene, which is contained in various plant substances, had effects on recovering the changes in metabolites occurring in high-fat diet (HFD)-induced obese C57BL/6 male mice brains, especially in the prefrontal cortex (PFC) and hippocampus (HIP). β-elemene also partially reversed HFD-induced changes in the composition and contents of mouse gut bacteria. Furthermore, we evaluated the interaction between cerebral metabolites and intestinal microbiota via Pearson correlations. The prediction results suggested that *Firmicutes* were possibly controlled by neuron integrity, cerebral inflammation, and neurotransmitters, and *Bacteroidetes* in mouse intestines might be related to cerebral aerobic respiration and the glucose cycle. Such results also implied that *Actinobacteria* probably affected cerebral energy metabolism. These findings suggested that β-elemene has regulatory effects on the imbalanced microbiota-gut-brain axis caused by obesity and, therefore, would contribute to the future study in on the interplay between cerebral metabolites from different brain regions and the intestinal microbiota of mice.

## 1. Introduction

Obesity is characterized as a kind of low-grade systematic inflammation with the potential to cause deficits in memory, behavior, or learning ability compared with a healthy state [1]. Higher concentrations of glutamine and glutamate in the hippocampus (HIP) have been reported when Wistar rats are subjected to high caloric intake with glucose intolerance [2]. The finding clarified the relationship between obesity and cerebral metabolites. Substantial neural circuitry and neuroendocrine activity disorders, impaired cortisol feedback, imbalanced neurotransmitter metabolism, alternating dopamine systems, and defective cognition are derived from obesity-related inflammatory processes in adipose tissue [3]. One of the intrinsic factors underlying obesity-related brain dysfunction is the gut microbiota, which seems to represent a link between environmental pressures, such as lifestyle and diet, and the host physiology that regulates neural pathways [4]. The microbiota-gut-brain axis contributes greatly to treating obesity-induced diseases in the central nervous system (CNS), including the prefrontal cortex (PFC), HIP, and hypothalamus (HYP). The PFC is the implementation center of the CNS, and damage to the PFC influences cognitive function, behavioral ability, and mental conditions in humans [5]. HIP is related to memory and emotion control in the brain. Furthermore, the stress response via the HYP-pituitary-adrenal axis is governed by the gut microbiota [6].

The intestine plays an important role in the exchange of environmental substances and organisms. Intestinal microorganisms are essential for promoting sugar and lipid metabolism and maintaining the body’s energy balance. The major intestinal microbiota members are divided into nine phyla: *Actinobacteria*, *Bacteroidetes*, *Deferribacteres*, *Firmicutes*, *Fusobacteria*, *Proteobacteria*, *Synergistetes*, *Tenericutes*, and *Verrucomicrobia* [7]. These microbiota are mainly involved in the synthesis of enteric short-chain fatty acids, of which acetate, propionate, and butyrate are considered to be closely related to obesity. Acetate stimulates the parasympathetic nervous system and then promotes the secretion of insulin and ghrelin to increase appetite [8]. In contrast, propionate and butyrate have anti-obesity effects [9]. In particular, butyrate is the main energy source for colonocytes and has a regulatory function in the integrity of the gut barrier. Therefore, butyrate exerts an anti-inflammatory effect on enteric innervation activity [10]. Sodium butyrate also has an antidepressant function by upregulating the expression of brain-derived neurotrophic factor, which plays an important role in inhibiting mood disorders [11]. This feedback is involved in the brain-gut axis and is essential for energy metabolism related to obesity.

Elemene is a sesquiterpenoid that is composed of α-elemene, β-elemene, δ-elemene, and γ-elemene [12]. These elemenes are first extracted from the genus *Zingiberaceae* as anticancer ingredients. Among them, β-elemene is more effective in anticancer effects than its isomers, while its conventional dose has no effect on other normal cells, such as peripheral blood leukocytes [13]. Recently, our study elucidated that β-elemene suppressed experimental obesity-induced chronic inflammation on adjusting the intestinal immune system of obese mice [14]. In addition, β-elemene passes through the blood–brain barrier, delays the onset of experimental autoimmune encephalomyelitis, and promotes the expansion of regulatory T cells in both the periphery and the inflamed spinal cord [15]. Although β-elemene was proved to mediate intestinal immune system and go through the blood–brain barrier to treat inflammation in the brain, it remains unclear whether β-elemene affects the gut-brain axis, which is the substance or information exchange feedback system between the gut and brain.

In this study, we explored the effects of β-elemene on alleviating obesity-induced imbalance of the microbiota-gut-brain axis. Nuclear magnetic resonance (NMR) and multivariate data analyses were performed to classify the changes in cerebral metabolites in the three CNS parts of the mouse brain, i.e., PFC, HIP and HYP, which execute behavioral, memorial, and hormonal regulation, respectively. Furthermore, the Pearson correlation was used to predict the interaction of the cerebral metabolites and intestinal microbiota.

## 2. Materials and Methods

### 2.1. Mice

C57BL/6 male mice (8 weeks old, weighing 20 ± 3 g) were purchased from Charles River Laboratories Japan (Yokohama, Japan) and were maintained at the appropriate temperature (23 ± 2 °C) and humidity (50 ± 5%), with a 12 h light/dark cycle. The mice were administered a normal diet (AIN-93G, Oriental Yeast Corporation, Tokyo, Japan) or diet with 60 kcal% fat (HFD-60, Oriental Yeast Corporation) separately for 12 weeks. Distilled water or water-dissolved β-elemene suspension (7.5 mg/kg/d; 0.2 mL) was administered by gavage for the last 3 weeks. The 3 mice groups were identified as follows, CON (normal diet), HFD (high-fat diet), and ELE (HFD-induced obese mice under treatment with β-elemene). A total of 12 mice (4 mice for each group) were used in the NMR experiments, and 9 mice (3 mice for each group) were used in the intestinal microbe test and Pearson Correlation calculation. The (-)-β-elemene analytical standard was purchased from Sigma-Aldrich (St. Louis, MO, USA). A detailed schedule is shown in Figure S1. All the experimental protocols were approved by the Experimental Animal Ethics Committee of the Graduate School of Agricultural and Life Sciences of the University of Tokyo (No. P19-026; approved on 17 May 2019). All procedures followed the Fundamental Guidelines for Proper Conduct of Animal Experiment and Related Activities in Academic Research Institutions under the jurisdiction of the Ministry of Education, Culture, Sports, Science and Technology, Japan. We have complied with all relevant ethical regulations.

### 2.2. Brain Sample Preparation

All dissected mouse brains were placed on ice. After washing with precooled PBS (-) (FUJIFILM Wako Pure Chemical Corporation, Osaka, Japan), 3 major regions of the mouse brain were separated: PFC (20 ± 3 mg), HIP (15 ± 5 mg), and HYP (10 ± 3 mg). The methanol (FUJIFILM Wako Pure Chemical Corporation): chloroform (FUJIFILM Wako Pure Chemical Corporation): Milli-Q water system with a volume ratio of 2:2:1 was selected as the extraction solvent of mouse brain tissue mentioned above. Using the ultrasonic bath-refining lyophilization method (300 W; 60 s) 2 times, polar metabolites in the mouse brain could be obtained [16,17]. For the NMR analysis, all reagents were prepared with deuterium oxide (D_2_O, Sigma-Aldrich). Metabolites were soaked in 630 μL D_2_O (PFC) or 225 μL D_2_O (HIP and HYP) after being freeze-dried, and 2,2-dimethyl-2-silapentane-5-sulfonate-*d*_6_ (DSS-*d*_6_, FUJIFILM Wako Pure Chemical Corporation) was then added to each PFC (70 μL), HIP and HYP (25 μL) sample at a final concentration of 0.5 mM as an internal standard. The metabolite concentration of each sample was standardized by tissue weight.

### 2.3. ^1^H NMR-Based Metabolite Analysis

^1^H NMR spectroscopy was performed on a Unity INOVA 600 spectrometer equipped with a cryogenic probe (Agilent Technologies, Ltd., Santa Clara, CA, USA) at 25°C. ^1^H NMR spectra were collected on 64 K data points over a spectral width of 8000 Hz, resulting in an acquisition time of 4.096 s. The delay time was set to 30 s, and water resonance was suppressed by presaturation. The free-induction decays (FIDs) were multiplied by an exponential function with a line-broadening factor of 0.3 Hz prior to Fourier transformation. The ^1^H signal of the methyl group of DSS-*d*_6_ was set to 0 pm and used as a standard signal for peak identification and quantitation of cerebral metabolites of mice with the help of Biological Magnetic Resonance Bank, MestReNova 11.0 (Mestrelab Research, Santiago de Compostela, Spain), Chenomx NMR Suite Professional 5.0 (Chenomx Inc., Edmonton, AB, Canada) [18] and some published papers [19,20]. To prove the difference in metabolites in the brain tissue of each mouse group, clustering analysis and principal component analysis (PCA) were performed using SPSS 22.0 (International Business Machines Corporation, Armonk, NY, USA) and SIMCA-P 13.0.3 (Umetrics, Umeå, Sweden).

### 2.4. Fecal DNA Extraction and Intestinal Microbiota Analysis

Mouse stool samples were frozen in liquid nitrogen before storage at -80 °C. Fecal pretreatment and DNA extraction were carried out according to a previous report [21]. The method of refining fecal DNA was PI-480 or NR-201 (Kurabo Industries, Osaka, Japan). A NanoDrop ND8000 (Thermo Fisher Scientific, Darmstadt, Germany) was applied to determine the concentration and purity of DNA. The primers were 341f/R806 (bacterial 16Sr DNA, approximately 430 bp) and dual-index (8-bp barcode). A polymerase chain reaction (PCR) assay was performed according to a previous method [21]. MiSeq (Illumina, San Diego, CA, USA) and MiSeq Reagent Kit v3 (Illumina) were used for unbound sequence analysis according to the manufacturer’s instructions. After connecting FASTQ using FASTQ-join [22], FASTX-Toolkit was used to screen QV (quality value) >20 in more than 99% sequences to execute the following experiments [23]. The deleted chimeras were determined by USEARCH 6.1.544_i86 using QIIME1.8.0 [24]. MultiExperiment Viewer (TM4 Inc., http://mev.tm4.org/#/welcome, accessed on 15 April 2020) was applied to plot the heat map, and Java was used to calculate the Pearson correlation.

### 2.5. Statistical Analysis

The results were presented as the mean ± standard error of the mean (SEM) and were analyzed by either one-way ANOVA followed by Dunnett’s multiple comparisons or two-way ANOVA followed by Dunnett’s multiple comparisons. A *p*-value <0.05 indicated significant differences.

## 3. Results

### 3.1. β-Elemene Regulated the Metabolite Contents of the PFC, HIP and HYP of Obese Mice

**Figure 1:**
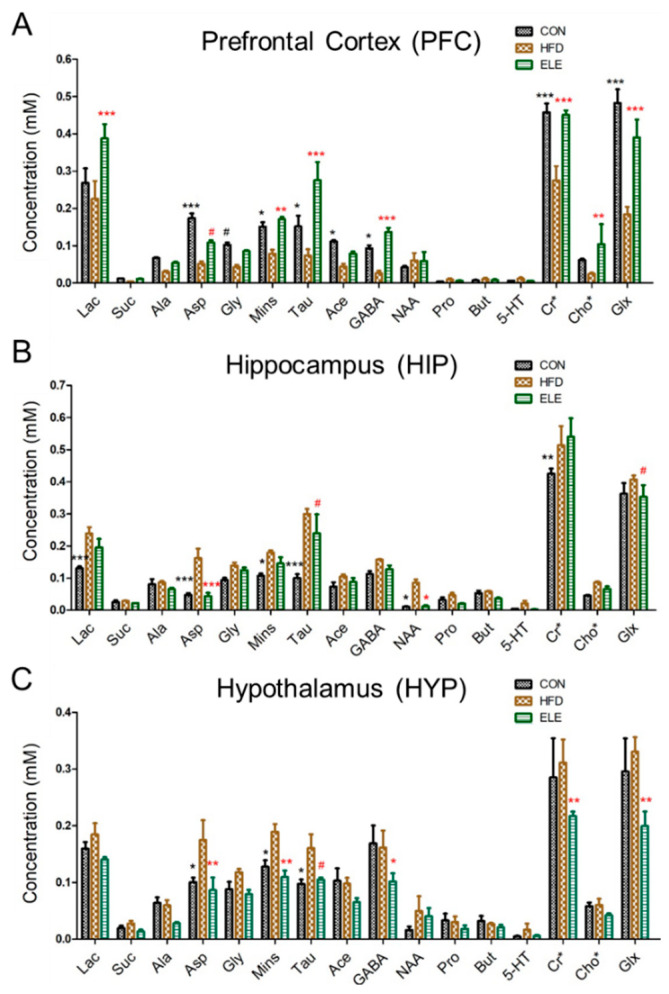
Comparison of mouse brain metabolites quantitated by ^1^H NMR spectroscopy. (**A**–**C**) Concentrations of cerebral metabolites in mouse prefrontal cortex (PFC) (**A**), hippocampus (HIP) (**B**), and hypothalamus (HYP) (**C**). CON, normal diet; HFD, high-fat diet; and ELE, HFD-induced obese mice under treatment with β-elemene. Cr*, Cr + PCr; Cho*, Cho + PCho; and Glx, Glu + Gln. The results are shown as the mean ± SEM (*n* = 4). ^#^ *p* < 0.1; * *p* < 0.05; ** *p* < 0.01; *** *p* < 0.001 vs. HFD group (assessed using two-way ANOVA followed by Dunnett’s multiple comparisons, the black color of * ^#^ means the differences between the CON and HFD and the red color of * ^#^ means the differences between the HFD and ELE).

For the obese mouse model (Figure S1), we found that β-elemene did not influence mouse body weight but alleviated the inflammation in the mice white adipose tissue [14]. The following cerebral metabolites, which are usually used to clarify the brain functions of mice, according to previous studies [19,25], were observed in the ^1^H NMR spectra of the PFC, HIP, and HYP in the CON, HFD, and ELE group: lactate (Lac), succinate (Suc), alanine (Ala), aspartate (Asp), glycine (Gly), myo-inositol (Mins), taurine (Tau), acetate (Ace), γ-aminobutyric acid (GABA), *N*-acetyl aspartate (NAA), propionate (Pro), butyrate (But), serotonin (5-HT), creatine (Cr), creatine phosphate (PCr), choline (Cho), choline phosphate (PCho), glutamate (Glu), and glutamine (Gln) (Table S1 and Figures S2–S4). As shown in Figure 1A, the PFC metabolites, including Asp, Gly, Mins, Tau, Ace, GABA, creatine + creatine phosphate (Cr + PCr, Cr*), and glutamate + glutamine (Glu + Gln, Glx), were decreased in the HFD group compared with the control group. Most of these changes were recovered by the oral administration of β-elemene. In addition, β-elemene increased Lac and choline + choline phosphate (Cho + PCho, Cho*) in the PFC. On the other hand, the concentrations of Suc, Pro, But, and 5-HT were low and hardly changed in the three groups. Figure 1B shows the normalized concentration of each metabolite of HIP from different groups. Compared with the control group, Lac, Asp, Mins, Tau, NAA, and Cr* were significantly increased in the HFD group. Among them, Asp, Tau and NAA were recovered by the oral administration of β-elemene. Similar to HIP, some metabolites of HYP were increased by the HFD treatment (Figure 1C), whereas β-elemene recovered Asp, Mins, and Tau to the level of the control group. In addition, GABA, Cr*, and Glx were decreased by β-elemene treatment.

### 3.2. Regulatory Effects of β-Elemene on Obesity-Induced Imbalance of Cerebral Metabolites Using Clustering Analysis and PCA 

**Figure 2:**
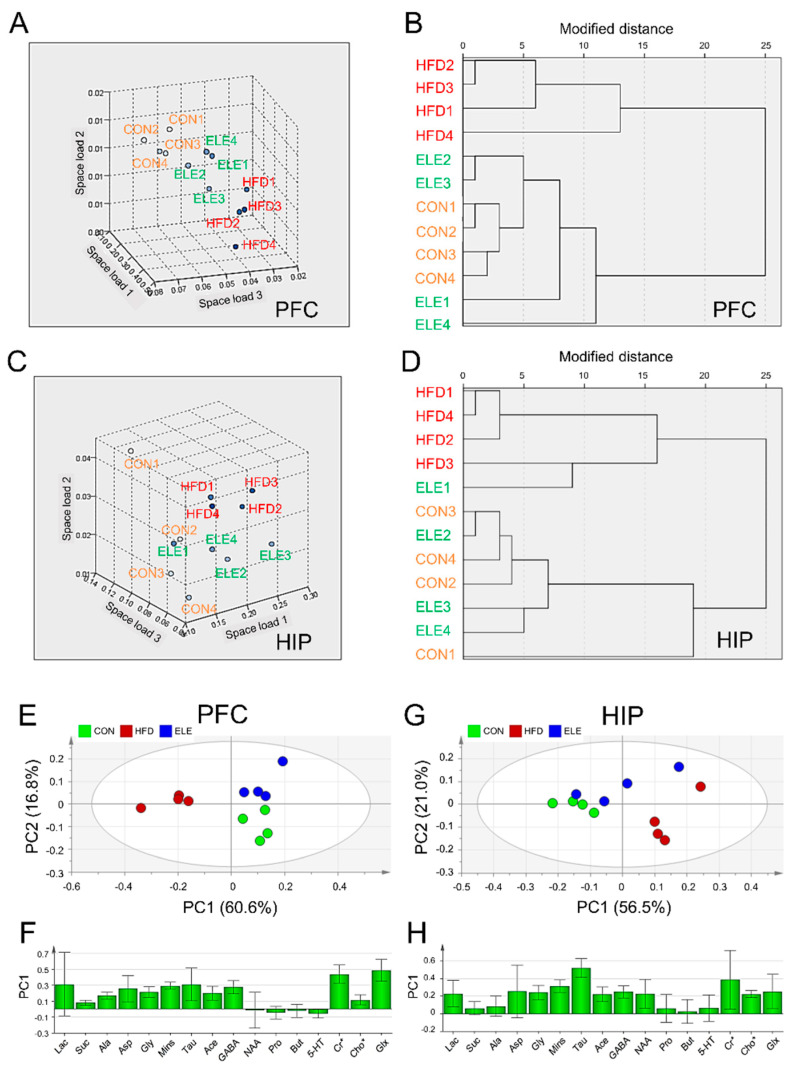
Cluster analysis and principal component analysis of mouse brain metabolites in different treatment groups. (**A**,**B**) Nearest neighbor analysis (**A**) and hierarchical cluster analysis (**B**) of PFC. (**C**,**D**) Nearest neighbor analysis (**C**) and hierarchical cluster analysis (**D**) of HIP. (**E**,**F**) Principal component analysis (PCA) score plot (**E**) and principal component 1 (PC1) loading plot (**F**) of PFC. (**G**,**H**) PCA score plot (**G**) and PC1 loading plot (**H**) of HIP. The spots with the same color are derived from the same kind of samples. CON, normal diet; HFD, high-fat diet; and ELE, HFD-induced obese mice under treatment with β-elemene (*n* = 4). Cr*, Cr + PCr; Cho*, Cho + PCho; and Glx, Glu + Gln.

Nearest neighbor analysis and hierarchical cluster analysis were used to further explore the effects of β-elemene on obesity-induced brain metabolite changes. The rotational space load of the ^1^H-NMR data of mouse PFC showed a distinct trend of separation between the HFD group and the other two groups (Figure 2A), and the load space distance between the ELE group and the CON group was smaller than that between the HFD group and CON group. Similarly, the ELE group and CON group were able to be gathered into one cluster, which was distinct from the HFD group (Figure 2B). These statistical data suggest the positive effects of β-elemene on obesity-induced PFC disorders with respect to cerebral metabolites. The two kinds of cluster analyses were also applied to classify HIP metabolites from different groups based on quantitative analysis data. A separation between the HFD group and the other two groups was observed in the rotational space load of the ^1^H NMR data of mouse HIP (Figure 2C), and the ELE group was clustered with the CON group separately from the HFD group (Figure 2D), which was a similar pattern to that of the PFC. In contrast, according to the clustering analyses of HYP, a distinct trend of separation was not observed among the three groups (Figure S5A,B).

PCA was applied to visualize the overall similarities and differences in cerebral metabolites among the three groups. The PCA score plot for PFC showed that the first two principal components (PCs) explained 60.6% (PC1) and 16.8% (PC2) of the total variance obtained for the three groups (Figure 2E). The HFD group was clearly separated from the other two groups on the PC1 axis. The positive factor loadings of PC1 indicated that the CON and ELE groups were characterized by higher amounts of GABA, Cr*, and Glx than the HFD group (Figure 2F). The PCA score plot for HIP showed that the first two PCs explained 56.5% (PC1) and 21.0% (PC2) of the total variance obtained for the three groups (Figure 2G). The HFD group was separated from the CON group on the PC1 axis but not the ELE group. The positive factor loadings of PC1 indicated that the HFD group had higher amounts of Mins, Tau, and NAA (Figure 2H). However, the HYP did not show clear separation among the three different groups (Figure S5C).

### 3.3. Effects of β-Elemene on Regulating Obesity-Induced Changes in the Composition of Intestinal Microbiota

**Figure 3:**
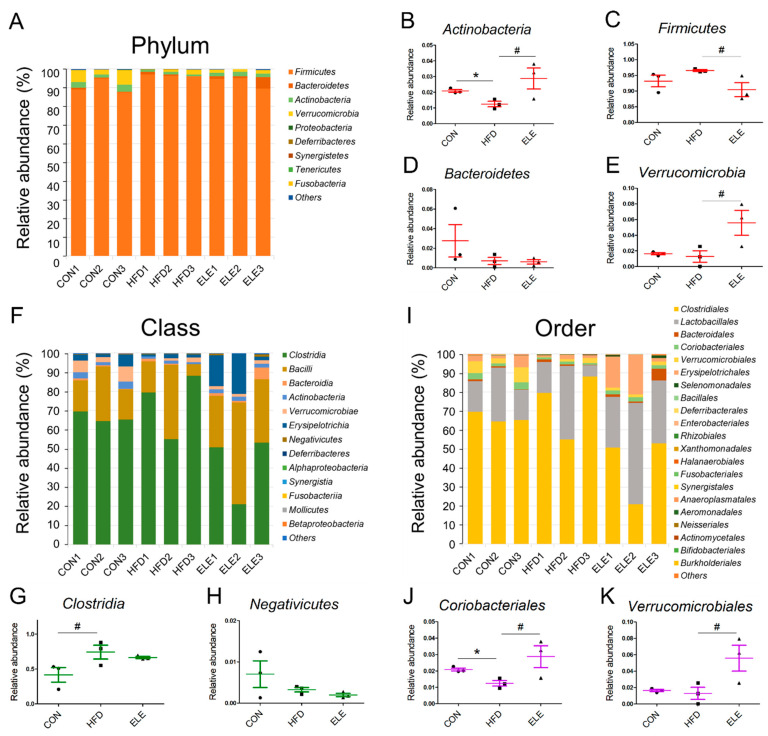
Relative abundance of phyla, classes and orders from intestinal microbiota in different treatment groups. (**A**) Relative abundance of all phyla. (**B**–**E**) Relative abundance of *Actinobacteria* (**B**), *Firmicutes* (**C**), *Bacteroidetes* (**D**), and *Verrucomicrobia* (**E**). (**F**) Relative abundance of all classes. (**G**,**H**) Relative abundance of *Clostridia* (**G**) and *Negativicutes* (**H**). (**I**) Relative abundance of all orders. (**J**,**K**) Relative abundance of *Clostridiales* (**J**) and *Verrucomicrobiales* (**K**). The results are shown as the mean ± SEM (*n* = 3). ^#^ *p* < 0.1; * *p* < 0.05 vs. the HFD group (assessed using one-way ANOVA followed by Dunnett’s multiple comparisons).

The four most abundant bacterial phyla were *Firmicutes*, *Bacteroidetes*, *Actinobacteria*, and *Verrucomicrobia*, and the minor phyla were *Proteobacteria*, *Deferribacteres*, *Synergistetes*, *Tenericutes*, and *Fusobacteria* (Figure 3A). Among the major intestinal bacteria, *Actinobacteria* was decreased in the obese mice but showed relative abundance at a level similar to that of the control group after 3 weeks of β-elemene treatment (Figure 3B). For healthy individuals, the *Firmicutes*/*Bacteroidetes* ratio was lower than that of obese individuals [26]. The proportions of Firmicutes and Bacteroidetes indicate the degree of dysbiosis in the gastrointestinal tract. In the present study, β-elemene decreased the relative abundance of Firmicutes in obese mice (Figure 3C) and did not affect the Bacteroidetes levels (Figure 3D). In addition, *Verrucomicrobia*, as a less abundant bacterial phylum, was proven to be downregulated by HFD treatment [27], and the β-elemene treatment increased the relative abundance of this beneficial microbiota (Figure 3E).

**Figure 4:**
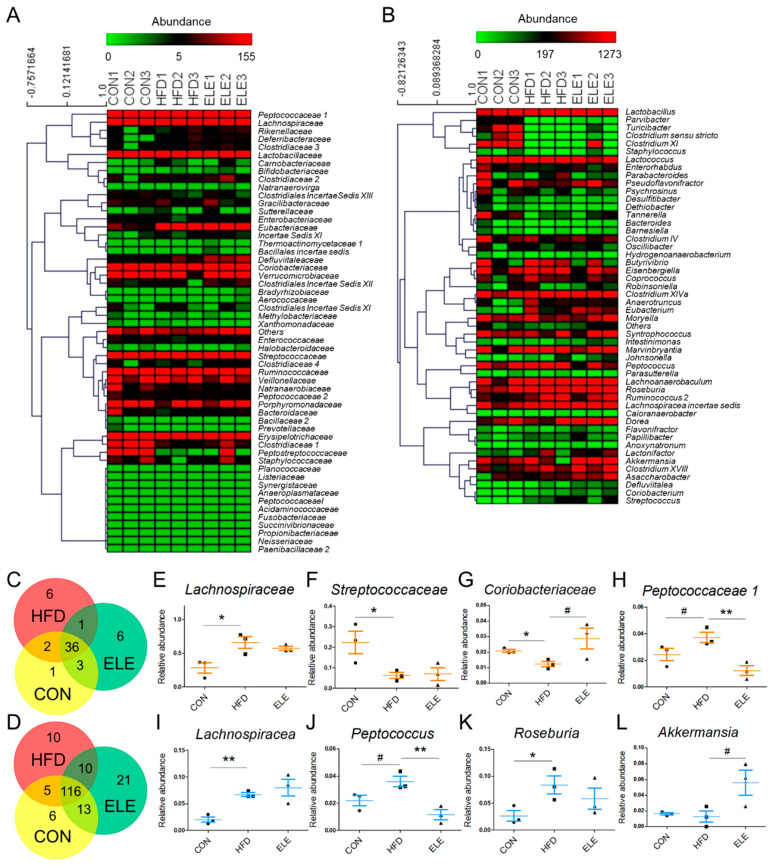
Relative abundance of families and genera from intestinal microbiota in different treatment groups. (**A**,**B**) Heat map of families (**A**) and the top 50 genera (**B**) with their relative abundances. (**C**,**D**) Venn diagram of families (**C**) and genera (**D**) in different treatment groups. (**E**–**L**) Relative abundances of *Lachnospiracea* (**E**), *Streptococcaceae* (**F**), *Coriobacteriaceae* (**G**), *Peptococcaceae1* (**H**), *Lachnospiraceae* (**I**), *Peptococcus* (**J**), *Roseburia* (**K**), and *Akkermansia* (**L**). The results are shown as the mean ± SEM (*n* = 3). ^#^ *p* < 0.1; * *p* < 0.05; ** *p* < 0.01 vs. HFD group (assessed using one-way ANOVA followed by Dunnett’s multiple comparisons).

To further explore the fecal microbiota of mice, deeper taxonomic levels, including at the class and order, were analyzed. The relative abundance of all the classes in the samples is shown in Figure 3F. *Clostridia*, *Bacilli*, *Bacteroidia*, *Actinobacteria*, *Verrucomicrobiae,* and *Erysipelotrichia* accounted for the relatively larger proportion (total >90%) of all detected classes. However, the abundance of *Clostridia* and *Negativicutes*, which belong to the phylum *Firmicutes,* was not reversed by β-elemene treatment (Figure 3G,H). At the order level (Figure 3I), the relative abundance of *Coriobacteriales*, which is an order belonging to *Actinobacteria*, was significantly reduced in the HFD group and recovered by the β-elemene treatment (Figure 3J). Moreover, increased tendencies of *Verrucomicrobiales* occurred after β-elemene intervention (Figure 3K). Furthermore, heat maps were used to visualize the large amounts of data from families and genera (Figure 4A,B). According to the Venn diagram in Figure 4C, there were 6 families specific to the ELE group, while it had the highest genus diversity among the three treatment groups (Figure 4D). There were differences in the relative abundance of some families and genera (Figure 4E–L). As shown in Figure 4E,F, HFDs increased the proportion of *Lachnospiracea* in obese mice, which was accompanied by a reduction in *Streptococcaceae*. In addition, HFD-reduced *Coriobacteriaceae* were increased after feeding β-elemene, whereas HFD-increased *Peptococcaceae 1* were decreased (Figure 4G,H). At the genus level, HFD feeding enhanced the proportions of *Lachnospiracea*, *Peptococcus*, and *Roseburia* (Figure 4I–K). Following the oral administration of β-elemene, a significant reduction in the relative abundance of *Peptococcus* and an increase in *Akkermansia* were observed (Figure 4J,L).

### 3.4. Cerebral Metabolites Correlated with Intestinal Microbiota

**Figure 5:**
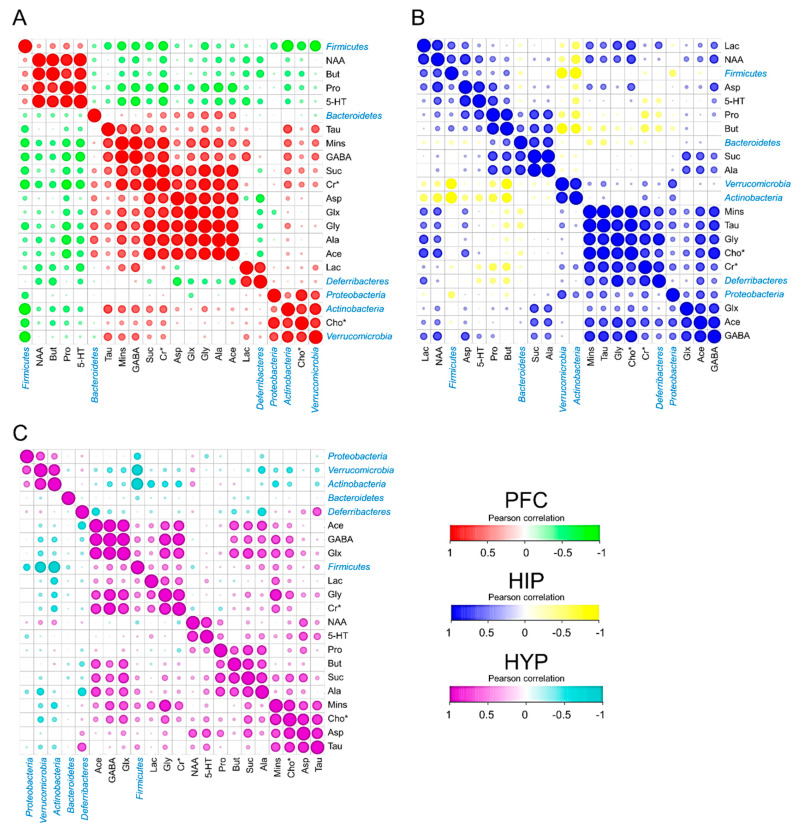
Cerebral metabolites correlated with the intestinal microbiota. (**A**–**C**) PFC (**A**), HIP (**B**), and HYP (**C**) metabolites correlated with intestinal microbiota (at the phylum level) in different treatment groups. Pearson correlation was used to calculate the correlation coefficient between cerebral metabolites and intestinal microbiota (*n* = 3). All the raw data were standardized from 0 to 1.

To explore the interaction of cerebral metabolites and intestinal microbiota, a Pearson correlation analysis was performed to calculate the correlation coefficients. The dominant phyla, including *Firmicutes*, *Verrucomicrobia*, *Actinobacteria*, *Bacteroidetes*, *Deferribacteres*, and *Proteobacteria*, and all the selected cerebral metabolites in the PFC, HIP, and HYP are visualized in Figure 5. All the absolute Pearson correlation values used in the following analysis were more than 0.45. In the PFC, *Firmicutes* had strong negative correlations with Mins, GABA, Cr*, Glx, and Cho*. *Bacteroidetes* showed higher positive correlations with Glx, Gly, and Ala. *Actinobacteria* seemed to be associated with Tau, Mins, GABA, Cr*, and Cho* (Figure 5A). In the mouse HIP, *Firmicutes* were the most abundant bacteria in the mouse intestine and corresponded to Lac and NAA. Both Suc and Ala showed highly positive correlations with *Bacteroidetes.* In addition, obvious negative correlations were observed between *Actinobacteria* and Lac, Pro, and But (Figure 5B). Similarly, as shown in Figure 5C, the relative abundance of *Firmicutes* varied based on the quantity of cerebral GABA, Glx, and Mins, and *Actinobacteria* was related to Lac and Cr*.

## 4. Discussion

The Microbiota-gut-brain axis is reported to contribute greatly to treating obesity-induced diseases in the CNS [4], which consists of PFC, HIP, and HYP. The three parts control the behavior, memory, and hormone secretion of mice, respectively [28]. In the present study, we found that the quantitative expressions of cerebral metabolites were significantly different among three brain regions of mice. Such as Mins and Tau, which attend neurodegeneration-associated inflammatory processes [29,30], represented a large decrease in the PFC, but a significant increase in the HIP and HYP of obese mice. However, the imbalance induced by the HFD was reversed by the β-elemene intervention. The results indicated that β-elemene normalized the effects of HFDs on various cerebral metabolites, although HFD-induced changes in the metabolites were region-dependent. In addition, to simplify the analysis when facing a large amount of data from the ^1^H NMR test, the PCA using SIMCA-P was applied to clarify the effects of β-elemene on treating the obesity-induced brain injury. The PCA results showed that CON and ELE groups were characterized by higher amounts of GABA, Cr*, and Glx than the HFD group in the PFC. GABA and Glx are paired metabolites that function as inhibitory and excitatory neurotransmitters, respectively [31]. The balance of these metabolites in the brain is the key point in promoting the normal proliferation and differentiation of neurons [32]. In addition, Cr* is considered to be present in regulating brain osmotic pressure and maintaining the stability of the brain cell membrane [33]. Therefore, β-elemene may be playing a role in neurotransmitter balance and osmoregulation of the PFC of obese mice. In the HIP, HFD group was clarified to have higher amounts of Mins, Tau, and NAA. Lizarbe et al. [34] clarified that increased Mins and Tau, which seem to be putative markers of inflammation, are responsible for hypothalamic inflammation in mice. NAA is sensitive to a series of pathologic processes of neuronal injury and axonal integrity. The concentration of NAA can reflect the number of neurons and their functional status [35]. However, the additional accumulation of NAA was considered a marker of risk in the HIP of obese mice [36], and a similar phenomenon was also shown in the present study as Figure 1B and Figure 2H. These findings highlight that normal brain function disorders and cerebral inflammation occur in the HIP of obese mice. 

Germ-free (GF) mice have increased plasma tryptophan, 5-HT, and hydroxyindoleacetic acid in HIP compared to normal mice [37], which clearly shows the relationships between intestinal microbiota and cerebral metabolites. After the quantitative calculations of mice brain metabolites, the composition of intestinal microbiota among the CON, HFD, and ELE groups at different biological microbiota levels was also analyzed in this study. For the phylum level, *Actinobacteria* was decreased in the HFD, but recovered by the β-elemene treatment. *Actinobacteria* is an important phylum for the maintenance of gut homeostasis [38], and it is responsible for inhibiting obesity in children and upregulated by intake of dietary fiber [39]. The relative order abundance of *Coriobacteriales*, which belong to the phylum *Actinobacteria*, was decreased by HFD feeding and reversed by β-elemene intervention. Similar results were also indicated by Soniwala et al. [40], who found that obesity reduces the order largely and hydrolyzed type 2 collagen showed excellent effects on alleviating the tendency and mitigating obese gut microbiome dysbiosis. At the genus level, an increasing tendency of *Akkermansia* was observed after β-elemene treatment. *Akkermansia* has been the subject of research interest, with Bodogai et al. [41] finding that the intestines of aged mice had significantly less *Akkermansia* than younger mice, and less *Akkermansia* triggered a decrease in butyrate, which is one of the key protective molecules of the intestine. At the same time, higher intestinal permeability and insulin resistance will occur as the circumstance continues. This advantageous bacterium was also upregulated by β-elemene intervention. The results mentioned above suggest that β-elemene regulates the composition and contents of fecal bacteria to partially mediate the imbalance of intestinal microorganisms in obese mice.

Furthermore, to define the relationships between mice cerebral metabolites and phylum microbiota under the HFD mouse model, their Pearson correlations with each other were then performed. *Firmicutes* were found to have a higher Pearson correlation with NAA, Mins, Glx, and GABA. NAA was clarified to affect the integrity of neurons, and Mins was reported as an inflammatory glial marker [42]. Both of them are increased in obese individuals [43]. Glx and GABA are important neurotransmitters [44]. Thus, *Firmicutes* were speculated to be involved in neuron integrity, cerebral inflammation, and the release of neurotransmitters. *Bacteroidetes* showed higher positive correlations with Suc, Ala, Gly, and Glx in the PFC and HIP. The evidence suggested that the changes in *Bacteroidetes* in mouse intestinal bacteria were associated with aerobic respiration in the brain and the glucose cycle. In addition, *Actinobacteria* had obvious correlations with Lac and Cr* in all three brain parts, which suggested that *Actinobacteria* may affect cerebral energy metabolism because Cr* promotes the release of adenosine triphosphate to prevent the accumulation of brain Lac [45]. Similar results have also been clarified based on the quantity and PCA and cluster analyses of cerebral metabolites according to ^1^H NMR spectra. These findings would contribute to the future study on the interplay between cerebral metabolites from different brain regions and the intestinal microbiota of mice.

## 5. Conclusions

In the present study, the effects of β-elemene on recovering HFD-induced changes in metabolites in the mouse brain, especially the PFC and HIP, were clarified and could be used to alleviate disorders of neuronal metabolism in obese mice. In addition, β-elemene reversed the HFD-induced changes in the composition and contents of obese mouse gut bacteria, although there are big differences in the abundance of individual intestinal microbiota between animals within one group. Finally, the interaction between the cerebral metabolites and intestinal microbiota of mice was determined based on a Pearson correlation analysis. These findings highlight the interplay between cerebral metabolites from different brain regions and the intestinal microbiota of mice, and they also suggested the potential effects of β-elemene on regulating the imbalanced microbiota-gut-brain axis caused by obesity.

## Data Availability

Data are contained within the article or supplementary material and are available on request from the corresponding authors.

## Supplementary Materials

The following are available online at https://www.mdpi.com/article/10.3390/biomedicines9070704/s1, Table S1: Chemical shifts of the metabolites, Figure S1: Schedule of the obesity mouse model, Figure S2: Representative ^1^H NMR spectra of the PFC of each mice group, Figure S3: Representative ^1^H NMR spectra of the HIP of each mice group, Figure S4: Representative ^1^H NMR spectra of the HYP of each mice group, Figure S5: Effect of β-elemene on regulating brain metabolites in obese mice based on cluster analysis and principal component analysis.
