# Supplementary material for: β-Elemene Suppresses Obesity-Induced Imbalance in the Microbiota-Gut-Brain Axis
Source: Biomedicines. 2021 Jun 22;9(7):704. doi: 10.3390/biomedicines9070704 (PMC8301405; doi:10.3390/biomedicines9070704)
Supplement: Supplementary file 1 [file biomedicines-09-00704-s001.zip › biomedicines-1232029-supplementary.pdf]

biomedicines

MDPI

Article

β-Elemene Suppresses Obesity-Induced Imbalance in the Microbiota-Gut-Brain Axis

Yingyu Zhou [1,2], Wanyi Qiu [1], Yimei Wang [1,2], Rong Wang [1,2], Tomohiro Takano [1,2], Xuyang Li [1], Zhangliang Zhu [1,3], Haruyo Nakajima-Adachi [2], Masaru Tanokura [1,2,*], Satoshi Hachimura [2,*] and Takuya Miyakawa [1,*]

Supplementary materials

Table S1. Chemical shifts of the metabolites.

Metabolite		^{1}H chemical shift (ppm)
1.	Lactate (Lac, $C_3H_6O_3$)	1.32, 4.10
2.	Succinate (Suc, $C_4H_6O_4$)	2.39
3.	Alanine (Ala, $C_3H_7NO_2$)	1.47, 3.77
4.	Aspartate (Asp, $C_4H_7NO_4$)	2.70, 2.79, 3.89
5.	Glycine (Gly, $C_2H_5NO_2$)	3.55
6.	Myo-Inositol (Mins, $C_6H_{12}O_6$)	3.26, 3.52, 3.61, 4.05
7.	Taurine (Tau, $C_2H_7NO_3S$)	3.25, 3.41
8.	Acetate (Ace, $C_2H_4O_2$)	1.91
9.	γ-Aminobutyric acid (GABA, $C_4H_9NO_2$)	1.89, 2.29，3.00
10.	*N*-acetyl aspartate (NAA, $C_6H_9NO_5$)	2.01, 2.48, 2.68, 4.38
11.	Propionate (Pro, $C_3H_6O_2$)	1.04, 2.17
12.	Butyrate (But, $C_4H_8O_2$)	0.88, 1.55, 2.14
13.	Serotonin (5-HT, $C_{10}H_{12}N_2O$)	3.11, 3.31, 6.88, 7.11, 7.29, 7.420, 10.00
14.	Creatine (Cr, $C_4H_9N_3O_2$)	3.02, 3.92
15.	Creatine phosphate (PCr, $C_4H_{10}N_3O_5P$)	3.03, 3.94
16.	Choline (Cho, $C_5H_{14}NO$)	3.20, 3.51, 4.05
17.	Choline phosphate (PCho, $C_4H_{10}N_3O_5P$)	3.21, 3.58, 4.16
18.	Glutamate (Glu, $C_5H_9NO_4$)	2.08, 2.34, 3.75
19.	Glutamine (Gln, $C_5H_{10}N_2O_3$)	2.13, 2.45

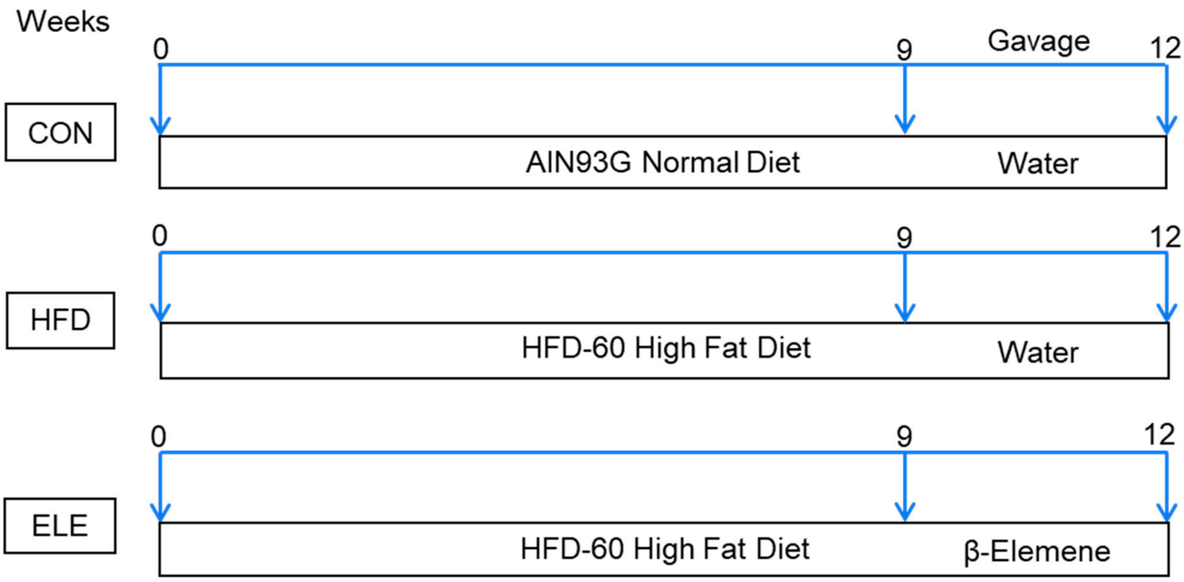

Figure S1. Schedule of the obesity mouse model.

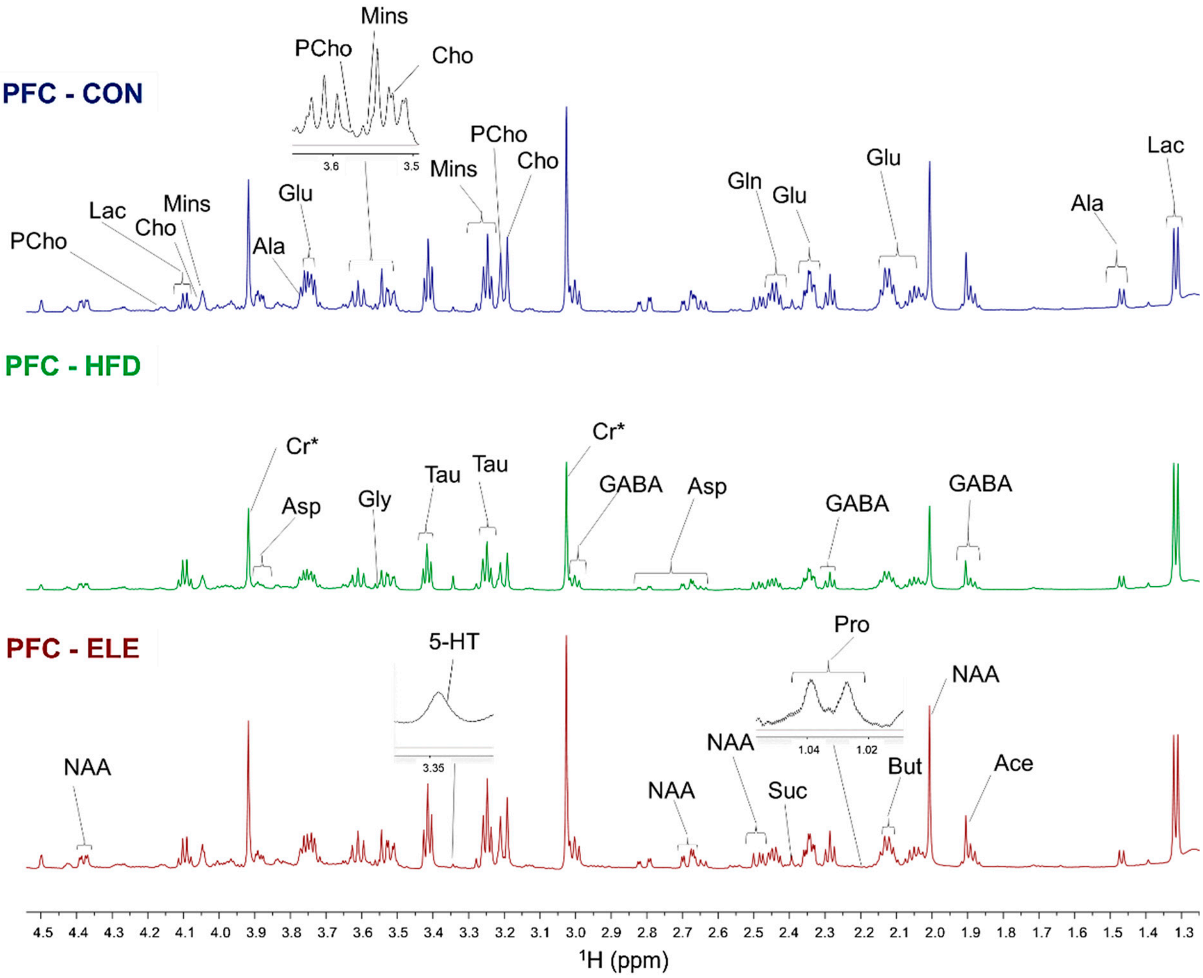

Figure S2. Representative ^{1}H NMR spectra of the PFC of each mice group. Cr*, Cr + PCr; CON, normal diet; HFD, high-fat diet; and ELE, HFD-induced obese mice under treatment with β-elemene.

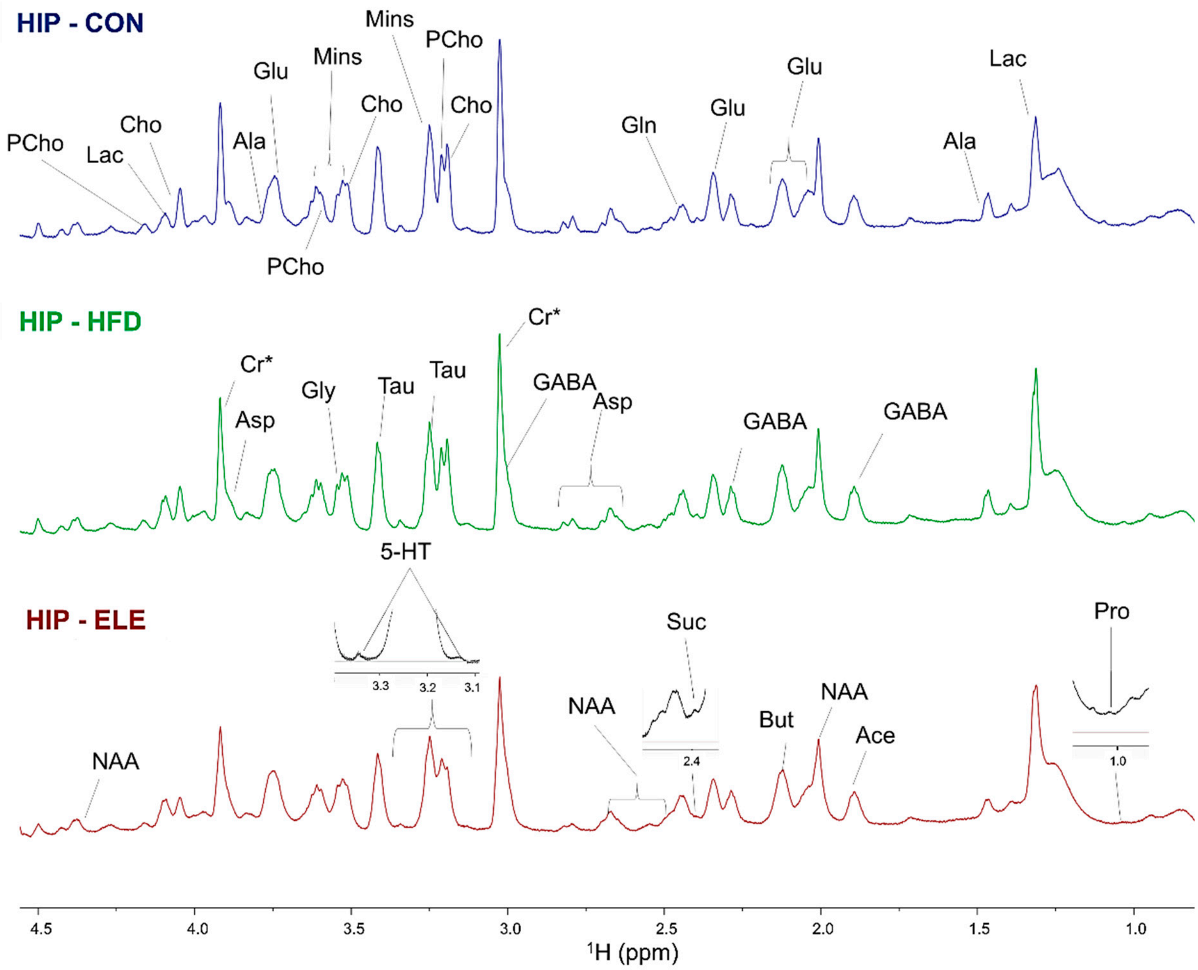

Figure S3. Representative ^{1}H NMR spectra of the HIP of each mice group. Cr*, Cr + PCr; CON; normal diet; HFD, high-fat diet; and ELE, HFD-induced obese mice under treatment with β-elemene.

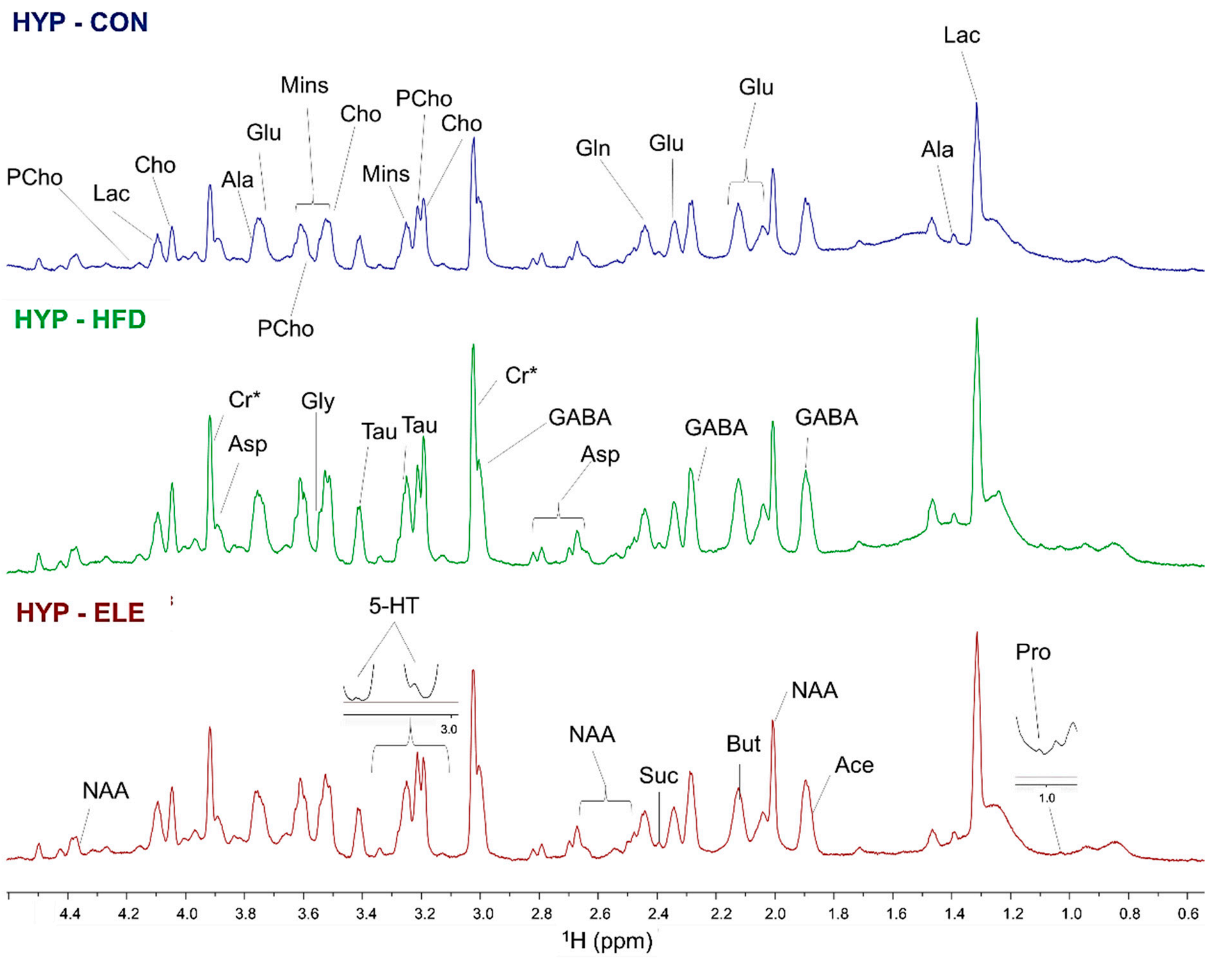

Figure S4. Representative ^{1}H NMR spectra of the HYP of each mice group. Cr*, Cr + PCr; CON, normal diet; HFD, high-fat diet; and ELE, HFD-induced obese mice under treatment with β-elemene.

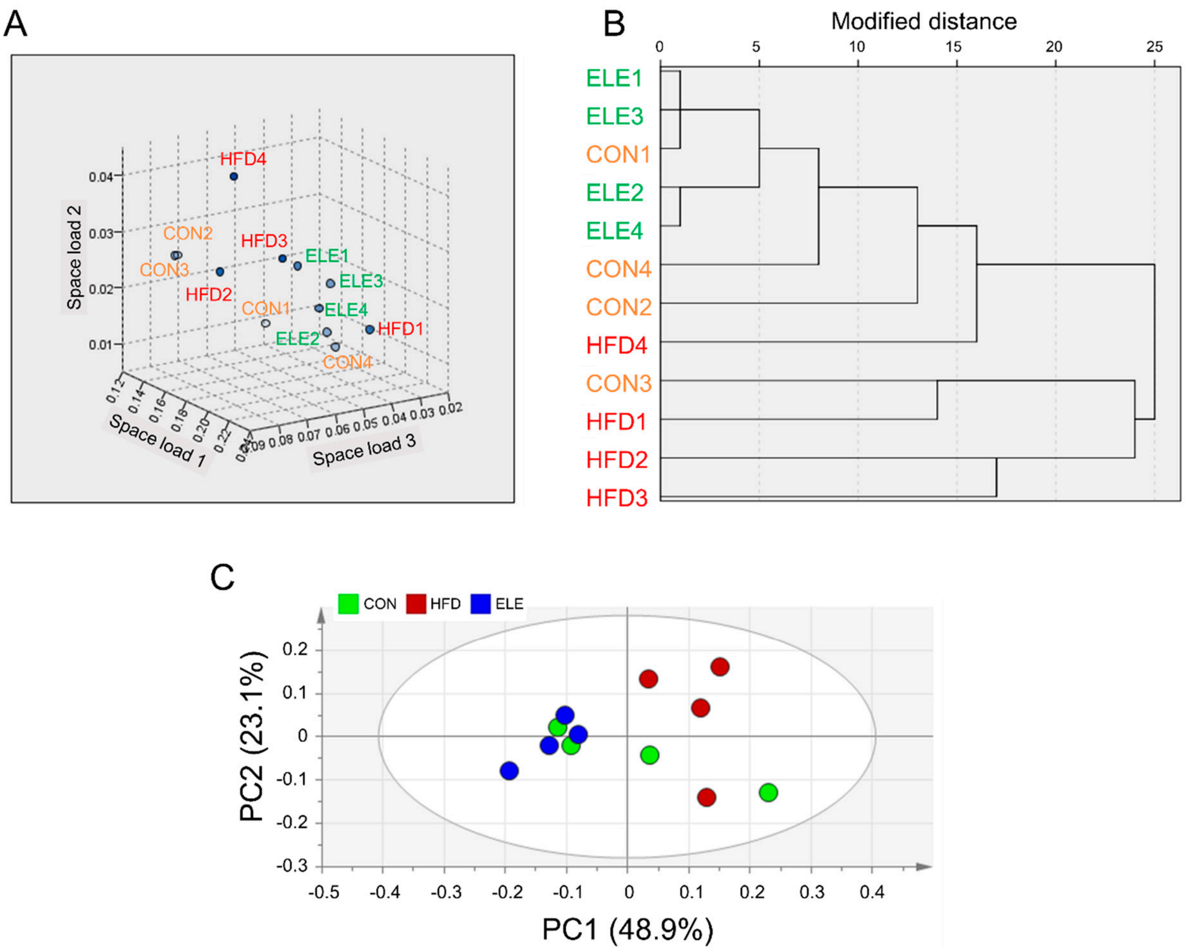

Figure S5. Effect of β-elemene on regulating brain metabolites in obese mice based on cluster analysis and principal component analysis. (**A**) Nearest neighbor analysis of the HYP. (**B**) Hierarchical cluster analysis of the HYP. (**C**) PCA score plot of the HYP. The spots with same color are derived from the same kind of samples. CON, normal diet; HFD, high-fat diet; and ELE, HFD-induced obese mice under treatment with β-elemene. Cr*, Cr + PCr; Cho*, Cho + PCho; and Glx, Glu + Gln.
